# *QuickStats:* Percentage* of Residential Care Communities^†^ That Use Electronic Health Records,^§^ by Census Region^¶^ — United States, 2016

**DOI:** 10.15585/mmwr.mm6725a8

**Published:** 2018-06-29

**Figure:**
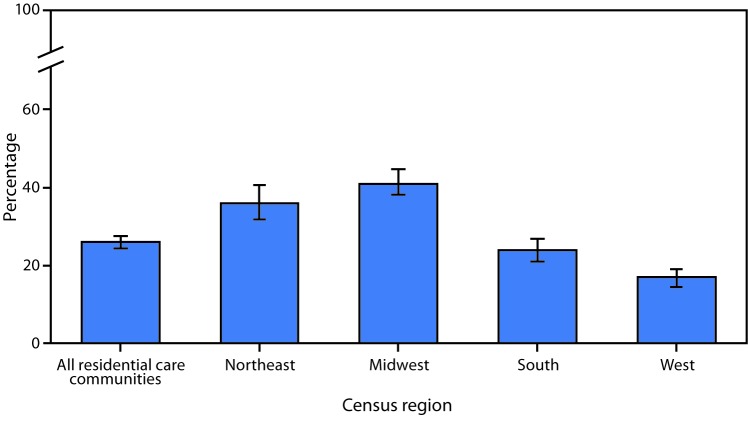
In 2016, 26% of residential care communities used electronic health records (EHRs). The percentage that used EHRs was 36% of communities in the Northeast, 41% of communities in the Midwest, 24% of communities in the South, and 17% of communities in the West.

**Source:** National Study of Long-Term Care Providers, 2016 data. https://www.cdc.gov/nchs/nsltcp/index.htm.

